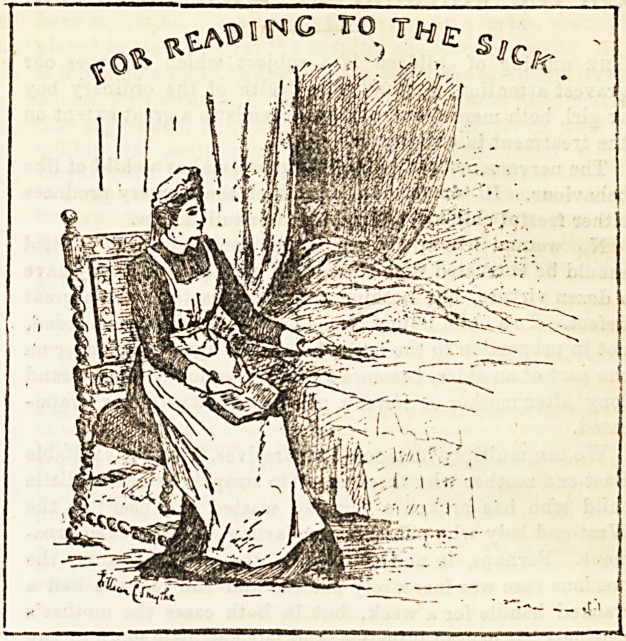# The Hospital Nursing Supplement

**Published:** 1891-11-14

**Authors:** 


### "The Hospital" Nursing Mirror.

Being the Extra Nursing Supplement of "The Hospital" Newspaper.

Contributions for this Supplement should be addressed to the Editor, The Hospital, 140, Strand, London, W.C., and should have the word "Nursing" plainly written in left-hand top corner of the envelope.

### En Passant.

YORK ROAD HOSPITAL.—We are glad to learn the six pupils sent up from this hospital have all successfully passed the London Obstetrical Society's Examination: Miss Olive A. Derry, Miss Eleanore Besson, Miss S. J. Lyon, Miss C. Garwood, Miss C. Malt, Miss E. A. Cox. Miss Derry sailed on the 29th of October for China. We wish her God speed in the hospital she is going out to as Matron. The other successful pupils are taking up private work.

GLASGOW NURSES.—We have received a letter from a lady in authority in Glasgow who, we are glad to state, says that the nurses at the Royal no longer look pale and tired, but cheerful and contented; we are heartily glad. Our correspondent says we have been too hard on the nurses; that they bore their trials as long as they could, and that she believes in future they will be wise enough if evils arise to go straight to the directors, who have done their very best throughout the late enquiry, and have remedied the faults that have existed too long. Let us hope that our next news from Glasgow will be in a more cheerful strain.

CLERGY CONFER ABOUT NURSES.—At the Hereford Diocesan Conference, the question of cottage nurses came up for discussion, but unfortunately the reverend members of the meeting seemed to think any woman could be a nurse, and they practically ignored the notion of training. Yet the clergy have to train for their profession. We advise the diocese of Hereford to affiliate with the rural branch of the Queen Victoria Institute and get good nurses; not to be content with little societies and half-trained "helps." We hope that the committee which has been formed will look at the question professionally. Amongst the remarks made were those by Mr. Sparrow, who thought the nurses instead of being located in the parishes should be located in the hospitals and infirmaries of the counties or town in the neighbourhood of their parishes. Mr. Askwith spoke of the work of the Hereford City Nursing Association, and thought the services of nurses should be free to the poor, and another reverend gentlemen urged that the Poor Law Guardians should facilitate Cottage Nursing, especially in midwifery cases, and pay the midwife a fee as they do the extra doctor's fee. Mr. C. D. Andrews called attention to the diffusion of knowledge that nurses might impart to the healthy as well as the unhealthy, and thought it desirable the nurse should be one who was duly qualified. The Rev. Davenport, with the consent of the Conference, read a paper on "Cottage Nursing," on behalf of Mr. Peter Giles, in which was mentioned the alternative plans of (1) Staff of nurses for village nursing. (2) Staff of nurses kept at a central home, such as a nursing institution or hospital, and sent out when required. (3) Organisation and education of present village-nursing women, supplemented by a small staff of hospital nurses to superintend nursing and take over cases requiring extra skill and care. The writer thought the first would be the most costly, that the second would work better, but the rich would get the benefit, and that the third was the cheapest and most easily worked. The writer also advocated the formation of a committee of clergy and laity to find out and register suitable women; also that medical men should hold an examination and award certificates to candidates.

IPSWICH NURSES' HOME.—The neat little annual report of this institution is pleasant reading; it tells of such wide work well carried out. The income last year was £1,234, and there is a small balance at the bank. Over 1,000 persons were nursed last year, some 200 bein[illegible] attended gratuitously, and 100 at reduced fees. Besides this t[illegible]e Home has supplied nurses to the Infectious Hospital, and to 33 of the surrounding villages. We congratulate the Committee and Miss Pye for their energetic and business-like methods of work.

WORKHOUSE NURSING.—The following report from the Medical Officer of the Keighley Workhouse was lately read to the Board of Guardians: "I find on visiting the infirmary to-day that the sick females have been left since mid-day on the 17th inst., without any nursing aid beyond the occasional assistance of the cook. The last-named official has this morning attended to the wants of the various foul cases, and would doubtless be called into requisition in the event of any infectious cases occurring. I must, therefore, call the attention of the Board to my recommendation of August 10th, and request them to re-arrange the nursing staff or add to its number without further delay." It seems that the only nurse having been granted four days' leave of absence to attend her father's funeral, the cook was left to superintend the nursing.

IRISH LUNATICS.—Ireland, weary land that she is, is behind in most things, not least in her care of the insane, as is shown in the report of the Special Commission. When the strong and healthy of a country emigrate, the weak left behind are out of due proportion, and, to save taxes, are not always well housed or cared for. The Commission recommends strongly that the insane poor should not be dealt with on a uniform system; that for the acute and curable cases there should be a hospital-asylum, with well-trained attendants and good medical attendance, and that those suffering from bodily sickness as well as mental derangement should be gathered in a separate section, and proper nursing provided. Again, they deprecate the keeping of harmless chronic cases in asylums, when they would be much happier boarded out. In truth, we agree with the Commission that the present Irish plan of herding all lunatics together in huge asylums, without system, is to be condemned.

SHORT ITEMS.—Our readers may remember that Miss Dewing, who is leaving the Eastern Hospital, had only been a few months at that institution when the disgraceful occurrences with regard to Nurse Halkin's departure took place. Nurse Halkin had been four years at the Eastern.—The fifth report of the Barton-on-Humber district nursing is satisfactory; Nurse Bennett has replaced Nurse Deas.—Rosa Francis was sentenced to two months' imprisonment last week for stealing from a late patient.—Miss Knight, Matron of the Adelaide, was one of the witnesses as to the filthy condition of children at the Carogh Orphanage.—A Vancouver paper describes the St. Luke's Home as much more flourishing since Sister Frances returned from England laden with gifts.—A Queen's nurse is now working in Inverness.—Cheltenham District Nurses have moved into a new home to meet their growing work; they appeal for a fund to furnish their larger premises.—The Bradford Nursing Society issues a good report; Nurse Bradley, of Birmingham, is now working for them.

### A National Pension Fund for American Nurses.

We have much pleasure in being able to state that a National Pension Fund for all workers among the sick in the United States has now been organised. Mr. Burdett has been spending some weeks in America and the authorities of various institutions took advantage of his being there to invite him to give an address in the chief Eastern cities with the object of testing public opinion, and, if thought desirable, securing the establishment of an American Pension Fund. Meetings were held during the last ten days of October at the Johns Hopkins Hospital, Baltimore; the University Hospital, Philadelphia; the hall of the Natural History Society, Boston; and in the chapel of the St. Luke's Hospital, New York. These meetings were supported by a large number of influential Americans, including the following: The President of the Johns Hopkins Hospital; Dr. Samuel Eliot, Chairman of the Trustees of the Massachusetts General Hospital; Bishop Potter, Bishop Phillips Brooks, the Rev. Dr. Greer, Messrs. J. Pierpont Morgan, Oliver W. Peabody, representative members of the medical profession, including Drs. Billings, Polk, Shattuck, Putnam, Pratt, Rowe, Chapman, Hurd, Cowles, and Worcester; the Matrons of all the principal hospitals; and many hundreds of nurses. The last meeting was held on the 27th ult., at St. Luke's Hospital, New York, under the presidency of the Rev. Dr. Baker, and was very largely attended. As the result a committee was appointed at each of these meetings to organise one scheme for the whole of the United States, and the Committee is now sitting in New York.

The actuaries of the Mutual Life Insurance Company of New York—the greatest insurance corporation of the United States—kindly prepared the necessary tables free of cost. It is satisfactory to note that the chief actuary, Mr. Emory McClintock, in a letter dated the 20th ult., addressed to Mr. Burdett, states: "I am satisfied, as an actuary, of the entire soundness, from a mathematical and scientific point of view, of the rates of the Royal National Pension Fund. I can say this positively, and from my own knowledge of the principal rates, viz., those for pensions. The rates for sick-pay assurance relate to a subject upon which I think there are no American data. As they are sanctioned, however, by so eminent an English actuary as Mr. King, I have no hesitation in approving them, at any rate, for present use, and until a large experience in both countries may suggest changes in detail. I take the greatest pleasure in expressing my hearty approval of the system pursued by the noble institution of which you are the founder."

It has been decided to organise the American Fund under the laws of the State of New York. These laws provide that 100,000 dols. (£20,000) must be invested as security to the policy-holders in the first instance, and this sum Mr. Burdett obtained from two gentlemen (Mr. J. Pierpont Morgan and Mr. W. O. Mills), each of whom provided 50,000 dols. to enable the Fund to be organised immediately. Mr. Pierpont Morgan has already contributed largely to the Royal National Pension Fund, and it is gratifying to be able to announce that he has consented to become the President of the American Fund. Mr. W. O. Mills is well known in New York, where he has contributed very handsomely to the Belle Vue Training School for Nurses, in which he takes the deepest interest. Subscriptions have been promised in each of the large cities, and there is reason to believe that Baltimore, Philadelphia, Boston, New York, and Chicago will each contribute at least 50,000 dols. After most careful consideration and consultation with the chief American authorities, Mr. Burdett has come to the conclusion that 250,000 dols. (£50,000) is the maximum sum which it is desirable to place to the credit of the American Fund from outside sources, apart from the payments of the members who join the Fund.

The Council of the American National Pension Fund will consist of the leading financiers of the six great Eastern cities, of representatives of (*a*) the Nurse Training Schools, and (*b*) the annuitants who place their savings in the Fund.

The utmost enthusiasm has been aroused in America on behalf of this movement, and before the end of the year it is expected that the American Fund will be actively working. Dr. Billings stated at the meeting on the 27th ult. that the authorities had determined to offer facilities which would probably bring in the whole of the Army nurses, numbering from seven to nine hundred. It is very gratifying to be able to report the institution of a National Fund for American Nurses, because by its establishment, having regard to the fact that the Royal National Pension Fund is capable of providing for the whole of the workers among the sick throughout the British Empire, every nurse or other worker amongst the sick who devotes his or her life to this work, and who is prepared to show reasonable thrift in any part of the English-speaking world, will henceforward be sure of adequate provision against sickness and old age.

Mr. Burdett's address will be published in a few days in pamphlet form, and copies may be obtained on application to the publisher of The Hospital, 140, Strand, W.C. The address contains many novel features of interest, and we regret we have not space to give it in our columns. The price has been fixed at 1s., so that anyone interested may readily obtain a copy for a nominal outlay.

It is our pleasant duty to record the invaluable services which have been rendered to the American Fund by Mr. J. Pierpont Morgan, Mr. W. O. Mills, Dr. Billings, Dr. Edward Cowles (of Boston), Dr. Chapman, Dr. Polk, the Rev. Dr. Baker, Dr. Hurd, Dr. Rowe, Dr. Samuel Eliot, the Rev. Dr. Fulton, Mr. F. B. Thurber, Mr. H. C. Meyer, Mr. Cunliffe Owen of the *New York Tribune*; Mr. G. W. Childs and Mr. Davies, of the *Public Ledger* (Philadelphia), Mr. Emory McClintock, and Mr. John Tatlock, jun., the assistant actuary of the Mutual Life Insurance Company, of New York. Mr. Tatlock not only prepared the necessary tables, but gave an immense amount of time to the organisation of the meeting at St. Luke's Hospital, which was one of the largest and most successful which has ever been held in connection with the Pension Fund movement from first to last.

The Committee already appointed, with power to add to its number, includes Bishop Potter, Mr. J. Pierpont Morgan, Mr. C. Vanderbilt, Dr. Billings, Dr. Polk, Dr. Hurd, Rev. Dr. Baker, Dr. Samuel Eliot, Mrs. W. P. Rogers, Dr. Cowles, Dr. Putnam, and Mr. Tatlock.

### Notes from Australia.

(*By Our Own Correspondent.*)

MELBOURNE, SEPTEMBER 5.

THE disgraceful business of electing the medical staff of the Melbourne Hospital by the votes of subscribers to the charity is finished; let us hope before another year comes round this state of affairs will have been altered. In the excitement of the poll the mother of one of the candidates dropped dead. The following letter to the *Argus* may be taken to show that disgust is felt at the present system:—

SIR,—Encouraged by your leading articles, and by the tone of the press generally, I have for some weeks waited to see the first move made towards a reform of the present mode of electing the medical officers of our general hospital. The evils of the present system of election are patent to all and must be galling to every self-respecting candidate.

Before this letter is in print the poll will be declared, and the right man may come to the top. Whatever be the result I should like to see a meeting of subscribers called for the purpose of ascertaining whether or not the subscribers as a body are in favour of retaining things as they are.

I have heard but one defence in the existing system—"it adds to the income of the hospital." If that is its only apology, I for one will cease to be a SUBSCRIBER.

The Cake Fair was a great success, and a cheque for £900 has been sent to the Melbourne District Nursing Society. There was a pin-cushion competition at the same time as the fair, and cakes and cushions vied in eccentricity.

The Charities Commission has been at Kyneton and Daylesford collecting evidence, but has come across nothing new, and there seems little to write about this month.

The Sisters of Nazareth, an order founded in London by Cardinal Wiseman, have lately opened a magnificent home for orphans and the aged poor at Ballarat. The building cost £14,000, and the Sisters will have to be good beggars to get their yearly income to maintain it.

At Sydney some excitement has been caused by the appearance of a vessel flying the yellow flag, part of the crew being down with small-pox. She is in quarantine at present and all the patients are recovering. I wonder do your readers know the story of the only ship that ever beat through the Straits of Gibraltar against wind and tide? She was coming from the Levant long long ago and got the black plague on board; she signalled the fact to the fleet, and the admiral gave orders to sink her and all on board. The methods of disinfecting were severe in those days. The captain rebelled and set all sail, chased by two of the fleet, he managed to get through the Straits and away to one of the West Indian isles, where most of the crew recovered, and after many years were taken back to England by a passing vessel. What a subject this for a poem by Tennyson!

### Appointments.

[It is requested that successful candidates will send a copy of their applications and testimonials, with date of election, to THE EDITOR, The Lodge, Porchester Square, W.]

BLACKBURN INFIRMARY.—Miss Susan Hale, who for the last twelve years has been Matron at Rotherham, has been elected Lady Superintendent of the Blackburn Infirmary. We congratulate the Blackburn authorities on their choice, for Miss Hale is an efficient Matron and a pleasant fellow-worker.

INDIAN SERVICE.—Miss Florence Walton, who trained at Westminster, has been appointed a Sister in the Indian Nursing Service, and sails on December 12th.

ST. JOHN'S HOSPITAL.—Miss E. J. Law has been appointed Sister-in-charge of this hospital in Leicester Square. Miss Law has worked at Cheltenham and East Dulwich Infirmary; she holds an honorary certificate from the London Hospital, where she at present holds the position of Staff-nurse Prince.

TYRRELL COTTAGE HOSPITAL.—Miss Hotson, who trained at the Manchester Royal, and lately acted as staff-nurse at Pendlebury, has been appointed Matron of this hospital.

### POLISHING.

THERE is no comparison, either for use or for beauty, between stones just dug out of a quarry and those built up into a fair and stately minster. The former are rough, shapeless, unsightly, the others smooth, even, and fitting properly into their appointed places. Not that they have lost aught of their natural value in the preparation, but simply their rough edges have been rounded off and their good qualities developed till they are worthy to be part of a noble building, a joy of the whole earth. Ere they arrive at this perfection, however, they have passed through many changes. Saw and hammer, chisel and plane have been brought to bear upon them, more or less, as the hardness and value of the stone will admit.

Let us look at those massive columns, those clustered shafts rising to support the graceful arches which, again, bear the ponderous roof above! Examine the exquisite carving of fruit and flower and berry and leaf which adorn them. Were these stones ever rude and uncouth masses covered with mould and dirt? Truly, yes; the Master Mason saw their value, and had them purified, carved, and polished, and now they are meet to form part of the house of the Lord.

In like manner, is it so with ourselves. God has designed a grand temple for His glory and service, and we as living stones must be shaped by trials and afflictions and sickness, and brought into conformity with His plan. Christ, the chief corner stone, was made perfect through suffering; shall we complain if our training be like His?

We may be a long while before we see the necessity for the pains and tribulations which we have to bear, the thwarting of our wills, the sufferings in the flesh, the rending of our natural affections, but we shall see hereafter in heaven where all things are made plain. St. Paul could say, and other Christians have echoed his words, "I reckon that the sufferings of this present world are not worthy to be compared to the glory which shall be revealed in us."

As day by day passes, our hearts are moulded and bent into uniformity with God's will. Our hasty tempers become chastened, our discontent changed to cheerfulness, and our waywardness to resignation. Our own infirmities will give us sympathy for those of others, and we shall be ready to bear one another's burdens as Christ bore ours. The Heavenly Architect saw that we wanted the polish which only suffering could give. He loves us so well that He could not bear to lose us from His blessed Salem. Of us it may be said that:

"Many a blow and biting sculpture,
Polished well those stones elect,
In their places now compacted
By the Heavenly Architect,
Who therewith hath willed for ever
That His Palace should be decked."

### On the Nursing of Sick Children.

The nursing of children is a subject which deserves our gravest attention, for the future health of the ordinary boy or girl, both mental and bodily, depends to a great extent on the treatment in childhood.

The nervous mother or fidgetty nurse makes a child of like behaviour. Ill-temper in the head of the nursery produces either fretful children or frightened or sullen ones.

No woman whose temper is not under perfect control should be entrusted with the care of children. She may have a dozen virtues, but nothing can compensate for this great defect. To a child unjust blame or the punishment awarded, not in proportion to the fault, but to the amount of anger on the part of an elder, becomes an injury which leaves a wound long after mother or nurse's momentary wrath has evaporated.

We can multiply instances for ourselves, from the excitable East-end mother who threatens "to strap" the grubby little child who has broken a jug and wasted the beer! to the West-end lady who punishes her darling for a broken ornament. Perhaps, in neither case is the child in fault: the precious vase was insecurely placed, and the jug has had a cracked handle for a week, but in both cases the mother's crossness has caused injustice, and left her little more to say when a real fault which merits correction is committed. Granting that children in ordinary health ought to be so carefully dealt with, how much more must this be urged in the treatment of sick children?

Infinite patience, cheerful temper, and unceasing watchfulness are needed in nursing them. Everything must be observed and accurately noted, for these patients cannot help us by describing their own symptoms. A child is seldom able to tell where a pain is; the presence of pain is confusing to its small mind, and it has a general feeling of misery which it confounds with the seat of the pain itself, and so points vaguely at a wrong spot.

Thus it is by the nurse's own observations that the truth is most quickly arrived at; she sees the rapid changes which take place often from hour to hour, and she will understand that nothing is too small to be noticed whilst studying this most delicate bit of machinery.

She will need all her natural tact and all her trained skill when the illness is long. Having gained the liking of the child, she will soon, if she be a fit and proper nurse, gain also its entire confidence; and a child's confidence is of a most complete character. It trusts so perfectly that the details of treatment are accepted because "nurse says so."

The medicine and food must be given judiciously, and if any one thing is really distasteful, after fair trials in varied forms, it should be mentioned at the doctor's next visit. Without giving way to all "fancies," any trifle which irritates the suffering child should not be persisted in.

To the regular performance of such duties as changing sheets, washing, &c., the patient soon becomes accustomed and speedily rewards the trouble taken for it by the contented little sigh with which a feverish child turns on its cool, fresh pillow.

The word "feverish" naturally leads us to the thermometer and to the extraordinary temperatures to which children attain, sending the mercury up or down in a most unexpected fashion very often. Even when the doctor only requires the temperature to be charted twice a-day, it is a very good plan for a nurse to take it more frequently, always at regular intervals, and to keep a record for her own instruction.

A nurse's note-book may prove really useful, provided that she is accurate and punctual in her records.

The washing of sick children is a most essential part of their treatment, and when a bath is ordered, every precaution must be taken to avoid chill; and when the water is ready (at the correct temperature), the fresh night-gown at the fire, and a blanket and towel also warming, the child should be quietly and gently placed in the bath, well screened round from all possibility of draughts, and itself covered carefully with a blanket.

The nurse must remain in close attendance until it is time to return the little patient to bed; it would be an error to leave the bath for a moment, and great care must be taken not to exceed the time for which it was ordered by the doctor.

If the child is well enough to sit well-wrapped up in a chair before he has his bath, nurse could rapidly arrange the bed for his return; but this must never be attempted after he is taken out of the bath, but assistance must be arranged for.

Washing in bed needs more ingenuity on the nurse's part, and she should spare no pains to make the process refreshing and thorough, and to avoid fatigue to the patient.

The night dress being quietly removed, and an enveloping blanket substituted, each part of the body should be washed with warm soap and water, and each part quickly dried and covered before the next portion is commenced on.

Many a "relapse" might be avoided if more care were taken on these points, and many a patient would have a better night if he were rapidly sponged all over just before his last evening meal.

If an energetic nurse beginning on a nervous child's face gets the water into his eyes she makes his ablutions a grief to him and a weariness to herself; but by commencing with the hands, and diverting his thoughts a little, he will probably consent to his face being washed quite cheerfully afterwards.

A frequent mistake in nursing children is the repetition of a command to "lie still" and "be quiet"; except to a very moderate degree this is inadmissible. As well tell birds not to sing, or kittens not to play! A child must move, and when he fidgets there is generally a very good reason, which it is the nurse's duty to find out and, if possible, to remove.

When a sick child is awake and lies quite still for any length of time it generally denotes weakness.

The different cries of little children should be noted as carefully as the character of a cough or the character and duration of the sleep taken.

To observe silently as well as quickly is necessary, for when a child knows itself to be watched it seldom acts naturally, but begins to feign, often unconsciously, but, of course, undesirably.

When a little patient is very weak there should be special accuracy observed in reporting the amount of nourishment taken—actually swallowed, I mean; and the nurse will need all her skill to coax down the proper amount when there is so little appetite for it.

Medicines are occasionally a difficulty at first, especially if the child has previously been cheated into taking an unpleasant dose by the assurance that it is "something nice." So obviously foolish a plan should never be tried, although a little reward after the medicine has been swallowed, or even a little "something nice" to take away the taste, is another matter entirely.

It should not be hastily decided that a child is "very naughty and disobedient" because he will not obey the nurse; probably, he has not been taught any discipline, and it cannot be learnt in a moment, but only let nurse exercise a little patience, and she will prove to him that she is worthy to be trusted and obeyed.

As regards "applications," it needs to be constantly borne in mind that children can endure both heat and cold in a far lesser degree than adults. Their skin is so delicate that the fomentation or poultice suited to a grown person would be most improper for them, and a source not only of danger but of positive injury, and great care must be taken when these applications have to be frequently repeated.

A nurse should comprehend that when a child-patient is not comfortable, not fairly good, or does not make satisfactory progress, the fact reflects discredit on her; it is her duty, as it should be her pleasure, to study the temper as well as the disease of her little charge.

Nothing is small, as regards a child, for every trifle affects its delicate organisation, and what seems a very little thing may retard its recovery.

To compensate for the multitude of its requirements we have the pleasant fact that a child is the best of all patients as regards a rapid convalescence and a complete restoration to health.

There is one point bearing on the nursing of sick children which must not be lost sight of in either hospital or home nursing—the child is the property of his parents. They may be his best friends and, alas, they may be his worst, but they are his parents with power to help or hinder the nurse to no inconsiderable extent. In facing the fact we must, when necessary, make the best of it. The wise father or mother is a valuable helper, and even the foolish ones can sometimes be persuaded to lay aside folly for the sick child's sake.

### Everybody's Opinion.

[*Correspondence on all subjects is invited, but we cannot in any way be responsible for the opinions expressed by our correspondents. No communications can be entertained if the name and address of the correspondent is not given, or unless one side of the paper only be written on.*]

### LOCAL HIGH TEMPERATURE.

"Lady Superintendent" writes: Can anyone help me to substantiate my notes on a case of tubercular peritonitis I have in my charge now, by finding out if anyone has noted a local temperature of 109 deg. and over? A temperature of 109·4 deg. was charted three days at different hours, in a four-hour chart, on absolutely undisputable authority, and two thermometers, taken in groin. But it seemed a local temperature. The pain was terribly acute in lower abdomen. As it was most unfortunately not taken simultaneously in other places, anyone's experience of such an occurrence would be most welcome. The patient is still alive, and, after repeatedly vomiting green matter, the pain ceased in that place and the temperature sank to sub-normal, only rising in groin twice again, once to 107 deg. It was then low in axilla and mouth. There is cystitis, &c. Facts so strange hardly appear true.

### NURSING ARRANGEMENTS AT THE METROPOLITAN HOSPITAL.

Esther J. A. Gilbert writes: May I call your attention to a misstatement of which, we are sure, you must be unaware, in your impression of the 5th, and ask you kindly to insert the following in your next? The Sisters of All Saints, with the Nurse of St. John's House, Norfolk Street, Strand, continue to nurse the Metropolitan Hospital, Kingsland Road, N.E., with perfect satisfaction to the medical staff and the Committee, as they have done since November 1st, 1888, with the sole exception that the Sister Superior does not sleep under its roof, as she is assisted by a Deputy Matron. These inaccuracies cause much extra work and inconvenience.

### PROPOSED MEMORIAL TO THE LATE MISS FREEMAN.

May we beg you to kindly allow us, through your widely-read paper, to bring to the notice of those many nurses who were trained at the Royal County Hospital, Winchester, under the late Miss Freeman, and who cannot fail to remember her with regard and gratitude, that it has been suggested a small memorial, such as a brass, should be placed in the chapel of the hospital she loved so well. As it is impossible for us to discover the addresses of all her former nurses, we venture to hope you will kindly assist us in making this suggestion known. Will any who approve, and are willing to contribute, communicate with Miss Gwyn, Royal Bath Hospital, Harrogate, before November 25th?—We are, Sir, yours faithfully, Christina Forrest (Lady Superintendent, County Hospital, York), Julia Birmingham (late Matron, Hospital, Bromley, and D'Arcy College, Winchester), Anna A. Gwyn (Royal Bath Hospital, Harrogate.

### NURSES AS PATIENTS.

"S." writes: I was glad to read "Nurse L.'s" letter testifying to the kindness which she had received in the London Hospital. I was not in the "London," but I, too, had received every kindness it were possible to have in St. George's Hospital (1869) when I was a patient there for nine weeks with scarlet fever. This before I had taken up nursing as a profession. I spent Christmas in the hospital, one of the happiest of my life. I must confess my recent experiences were a sorrowful contrast to my previous one of hospital life. It surprised me the more as one hears so much at the present time about the progress of nurses and the training they receive. Without wishing to be ungrateful to the kind surgeon who performed my operation most skilfully, and with perfect success, I am bound to say that every patient in the ward I was in was made painfully conscious of the fact that there was a certain work to be got through within a given time. This was usually accomplished with such a bustle, making the patients feel that there was no regard for any want beyond general routine.

### Christmas Competitions.

In answer to some of our correspondents we repeat the rules for these competitions, parcels for which must reach this office by December 12th. To secure warm garments for the adults in hospital on Christmas Day we offer the following prizes, which will be awarded in books or money as the winners choose: (1) For the best pair of socks knitted by a nurse, 5s.; (2) for the best pair of socks knitted by any Hospital reader, 5s.; (3) for the best made flannel shirt, 10s.; (4) for the best made woman's blouse, 10s.; (5) for the best made flannel petticoat, 10s.; (6) for the best made and best shaped dressing gown for an invalid cut out and made by a nurse, 20s. It will be seen that No. 1 and 6 are reserved for nurses only. With regard to No. 6 we specially hope for many entries, and if we secure them we propose to give more than one prize. Flannelette is cheap, and light, and warm, and would, therefore, form the best material for the dressing gown. In judging, four marks are given for workmanship, four for shape, and two for general appearance; therefore, it is not wise to spend time on elaborate trimmings. Long seams may be done by machine. Parcels of clothing for distribution, but not for competition, will be gladly received and acknowledged in these pages.

### Notes and Queries.

To Correspondents.—1. Questions or answers may be written on post-cards. 2. Advertisements in disguise are inadmissible. 3. In answering a query please quote the number. 4. A private answer can only be sent in urgent cases, and then a stamped addressed envelope must be enclosed. 5. Every communication must be accompanied by the writer's full name and address, not necessarily for publication. 6. Correspondents are requested to help their fellow nurses by answering such queries as they can.

### Answers.

*A. G.*—Thanks; but we cannot organise this question of nurses for the middle-classes just now; we have so much on hand. Tell the doctors in your neighbourhood that you are willing to take such cases.

(9).—If your chilblains are unbroken paint them with sulphurous acid. —*Doris.*

*Sister H.*—Apply to the Under-Secretary of State, India Office, Whitehall. We gave full particulars in two articles which appeared during August.

*Miss M. Maynard.*—You can use a *nom de plume* if you insert your proper name and address for our private knowledge. We do our best to answer all queries, save those asking us to prescribe.

*Agnes.*—We never prescribe; consult your medical attendant.

*Hospital Nurse.*—We are sorry, but we cannot give a second subject for competition. Surely you kept notes of cases during your training? Even in the out-patient department there should be cases well worth keeping note of.

*Nurse Lucy.*—We have made enquiries of the editor of the *Daily News*, and have come to the conclusion that it would be wiser not to carry out your generous proposal. The story was so very sensational, and we did not hear the whole of it.

*Christmas Competitions.*—Parcels received from Nurse Edith Gibson and Caroline Bartlett.

*Royal Albert Hospital.*—Your communication is anonymous, so we can make no use of it.

### Bruce.

*(Concluded.)*

At the same moment in which I made the discovery that the slowly moving red stream was blood—whose I knew not—Mary opened her eyes. With a gasp and then a shriek, she recognised that she was safe.

"Oh, thank God that you have come? He hasn't hurt me; but I think he has killed Bruce!"

It was a huge relief to find she was uninjured, and bidding her keep still I went across to the prostrate figures under the tree.

"Bruce, old boy, what is it?"

At my voice the dog's drooping head was raised; a great light of joy sprang into the dim eyes, and the mastiff's powerful grasp was relaxed. In dumb language it meant, "You've come at last, master. Take him over, and let me die!" and the noble head fell heavily. A sharp pain ran through my heart, for the meaning was so plain.

"My poor old man! Good old Bruce, what has he done to you?" Even as I spoke the words my eye caught sight of a cruel knife in the clenched human hand—a knife that had done deadly work, I feared. I also saw that its owner, an evil-looking tramp, was not scheming, but had swooned, probably in an agony of terror at the dog's attack.

"There you can lie," I muttered savagely, "until I look after your betters."

Tenderly I examined the noble creature that was dear to me as a child, for between Bruce and myself there existed an almost passionate attachment. I found what I dreaded. The mischief was irrevocable, and Bruce's hours were numbered. I bound up the most extensive of the wounds, and as I finished, help came in the shape of some villagers. Together we manufactured a rough and ready stretcher, and, after handing over the now conscious tramp to be conveyed to gaol, we carried our hero—Bruce—home, where Mary and I tended him unflaggingly for the short remainder of his span, feeding him with spoonfuls of beef-tea and brandy and water by turns. But it was all in vain; the ugly wounds inflicted by the tramp who had attacked Mary, and just succeeded in wrenching her watch from her when Bruce bounded upon him, were also vital ones; no earthly power could heal them. I knew too well that I watched beside what was the death-bed of a friend, as Bruce lay undisturbed on the stretcher, Mary's blue cloak wrapped round him, but powerless to warm the chills with which death was creeping on. Life was ebbing fast; and just when the night and the new day met face to face, Bruce stirred uneasily.

"What is it, old man?" I asked, with a choke in my throat, for I knew what was coming, and there was a low sob from Mary, as she read my face.

Raising his huge fore-paw, though it was a struggle to do it, Bruce groped about for something. Closer I bent over him, then the paw went into my hand, and Bruce was satisfied. Out from his glazing eyes there leaped a long look of love and farewell, and thus we parted. There are those who will calmly tell you that there is no hereafter for such as Bruce—for a hero who laid down his life as if it were an every-day duty to save his mistress.

Who is it has told us that: "Greater love hath no man than this, that a man lay down his life for his friends"? And, if a dog can emulate man's highest sacrifice, is it too much to hope that, be it spoken with all reverence, for him, too, there will be a place on that "other shore"?

We never had another dog, Mary and I; we pay that tribute to the memory of our faithful Bruce, and though we have left that little bit of our lives far behind in the background, we do not forget the modest grey stone, inscribed upon which is simply:

"For his friend;"

and which marks the spot, under the scented pines, where Bruce lies buried.

### A Bed for a Sick Nurse.

Since our last notice we have received the following sums, and we must again acknowledge our special indebtedness to Nurse Elms. Collected by Nurse Elms, £1 14s. 6d. (Mrs. John Webb, £1; Mrs. Cragg, 10s.; Mrs. Thomas, 4s. 6d.); Miss Mary Hudson, 5s.; Nurse Steer, 1s. 6d.; Nurse Anderson, 5s.; A Friend (through Nurse Anderson), 5s.; "Policy 24," 2s.; Miss Margaret Hunt, 2s. 6d.; Nurse Barbara, 5s.; A Friend (through Nurse Barbara), 5s.; making a total of £28 3s. 6d., and leaving only three more guinea subscriptions needed. Last week Mr. and Miss Webb were wrongly described as Welb.

### Presentations.

Mrs. Cann, who has been Matron of the Tyrrell Hospital since its foundation, has been presented by the governors and the medical staff with a retiring allowance and a handsome clock.

Miss Pidgeon, for five years at Hammerwich, has lately removed to the Jaffray Hospital. We are pleased to learn that before she left some slight acknowledgment of her valuable services was made by the Committee and others interested in the Hammerwich Cottage Hospital. A testimonial consisting of a handsome lady's dressing bag, with silver fittings and inscription plate, and a purse of 15 sovereigns, subscribed for by the Committee, medical staff, and patients of the hospital, was presented to her, and seldom, if ever, has such a testimonial been more richly deserved.

### Amusements and Relaxation.

### SPECIAL NOTICE TO CORRESPONDENTS.

**Fourth Quarterly Word Competition commenced October 3rd, ends December 26th, 1891.**

Competitors can enter for *all* quarterly competitions, but no competitor can take more than *one first prize* or *two* prizes of any kind during the year.

Proper names, abbreviations, foreign words, words of less than four letters, and repetitions are barred; plurals, and past and present participles of verbs, are allowed. Nuttall's *Standard* dictionary only to be used.

The word for dissection for this, the SEVENTH week of the quarter, being

"ORANGES."

| Names. | Nov. 5th. | Totals. | Names. | Nov. 5th. | Totals. |
|---|---|---|---|---|---|
| Lightowlers | 33 | 234 | Jenny Wren | 31 | 159 |
| Bonne | 33 | 256 | Darlington | 35 | 238 |
| Morico | 40 | 279 | Nurse G. P. | — | — |
| Robes | — | — | Hetty | 34 | 157 |
| Dulcamara | 34 | 245 | Janet | 24 | 198 |
| Psyche | — | — | Jackanapes | 32 | 177 |
| Agamemnon | 38 | 234 | Ex Nurse | — | — |
| Nurse J. S. | 36 | 239 | | | |

### Notice to Correspondents.

All letters referring to this page which do not arrive at **140, Strand, London, W.C.**, by the *first post on Thursdays*, and are not addressed PRIZE EDITOR, will in future be disqualified and disregarded.

N.B.—Each paper must be signed by the author with his or her real name and address. A *nom de plume* may be added if the writer does not desire to be referred to by us by his real name. In the case of all prize-winners, however, the real name and address will be published.

## Figures and Tables

**Figure f1:**